# Adderall-Induced Persistent Psychotic Disorder Managed With Long-Acting Injectable Haloperidol Decanoate

**DOI:** 10.7759/cureus.27273

**Published:** 2022-07-26

**Authors:** Saral Desai, Erika L Santos, Anca E Toma, Andrés A Henriquez, Adeel Anwar

**Affiliations:** 1 Department of Psychiatry, One Brooklyn Health, Brookdale University Hospital Medical Center, Brooklyn, USA

**Keywords:** adhd, attention deficit hyperactivity disorder, long-acting injectable, haloperidol, psychosis, misuse, stimulant, adderall

## Abstract

Adderall is one of the most commonly prescribed stimulant medications for attention deficit hyperactivity disorder (ADHD). Although safe and effective when clinically indicated at the appropriate dose, stimulant misuse may lead to serious adverse effects. We report a 29-year-old male with a diagnosis of ADHD who took more than the recommended therapeutic dose of Adderall prescribed by his psychiatrist. He subsequently presented with persistent psychotic symptoms, which responded to oral haloperidol. Due to treatment non-compliance with multiple recurring psychiatric hospitalizations, long-acting injectable haloperidol decanoate was considered to improve compliance and prognosis. The patient’s psychosis remained in remission while on the long-acting injectable. In this case study, we highlight the need for future research to identify stimulant misuse risk factors. Randomized clinical trials are needed to determine the effectiveness of long-acting injectable antipsychotic medication in the management of persistent psychosis secondary to stimulant misuse.

## Introduction

Adderall (mixed amphetamine salts) is one of the most commonly used stimulant drugs in the management of attention deficit hyperactivity disorder (ADHD). Stimulant medications such as Adderall can increase levels of the neurotransmitter dopamine in the brain, and over time lead to the phenomenon of “sensitization.” Amphetamines can mimic psychosis, especially when taken recreationally above the approved therapeutic doses. Stimulants when used in therapeutic dosage for treatment of ADHD have been shown to reduce the risk of substance abuse in these patient populations [1]. Additionally, there is imaging evidence to support that stimulant use among individuals with ADHD has differential effects as opposed to those without ADHD [2].

A recent meta-analysis suggests that childhood ADHD might increase the risk of developing subsequent psychotic disorders during adulthood [3]. There might also be a shared genetic susceptibility between childhood ADHD and adult schizophrenia [4]. Although, stimulant use for the treatment of ADHD is safe and effective, whether prolonged stimulant use/misuse contributes to the development of a subsequent psychotic disorder in genetically susceptible individuals remains unknown. Additionally, this relationship might be further complicated by overdiagnosis and treatment of ADHD as well as a rise in prescription stimulant misuse [5,6].

While acute amphetamine intoxication can present as a substance-induced psychotic disorder, some may go on to develop a psychotic disorder with onset during intoxication that persists [7]. In the young adult patient population, it poses a unique challenge in separating first onset psychosis from a stimulant-induced psychotic disorder and subsequent transition to schizophrenia-like illness, leading to diagnosis and treatment dilemmas. We describe such a case of a 29-year-old male with a stimulant-induced persistent psychotic disorder that responded well to oral haloperidol. In light of the patient’s recurrent hospitalizations and history of non-compliance to the treatment, a clinical decision was made to try a long-acting injectable for haloperidol decanoate.

## Case presentation

Mr. X is a 29-year-old Caucasian male who was brought to the emergency department (ED) for altered mental status. Mr. X was pulled over on the highway and was found to be agitated, incoherent, and responding to internal stimuli. Emergency medical services (EMS) were then called to bring Mr. X to the ED to be evaluated for altered mental status. Mr. X has a past psychiatric history of ADHD, anxiety disorder, and stimulant-induced psychosis. He has no known medical history. There is no known psychiatric history in Mr. X’s family. Mr. X is single and lives with his mother. Mr. X holds a bachelor's degree in computer science and plans to attend law school. Mr. X has no known history of illicit substance use. Mr. X was diagnosed with ADHD as an adult when he started college, and he was first prescribed Adderall at the age of 19 and has taken a dosage of up to 90 mg per day. Mr. X had been hospitalized twice in an inpatient psychiatric unit for a similar symptomatic presentation that was attributed to Adderall use above the approved therapeutic dosage (90 mg/day). During his past presentations, he was prescribed olanzapine and risperidone for the management of psychosis, but he was non-compliant with treatment for unspecified reasons. Following his last discharge from an inpatient unit, Mr. X continued to see different psychiatrists over the telemedicine platform and continued to obtain Adderall.

En route to the hospital, his blood pressure was noted to be 150/90 mmHg, with a pulse rate of 112 beats per minute, and he was given 10 mg of midazolam. The initial medical workup was negative, which included a negative head CT scan and infectious disease workup. Urine toxicology was not possible initially as Mr. X refused to give a urine sample. However, based on vitals, physical examination, and Mr. X's confession, it was determined that Mr. X took multiple pills of prescription stimulant Adderall before the presentation. Mr. X was soon medically cleared, and a psychiatric consult was placed for further evaluation. Upon initial psychiatric evaluation in the ED, Mr. X was noted to be guarded and had an intense stare with bloodshot eyes and dilated pupils. He displayed elevated mood, bizarre behavior, and grandiose delusions of being a supreme court judge. He was perseverating with legal terms and speaking circumstantially and rapidly. He was transferred to emergency psychiatry (Comprehensive Psychiatric Emergency Program (CPEP)) for further observation. In CPEP, Mr. X became more agitated, verbal de-escalation was unsuccessful, and he refused oral medications. He was given intramuscular medications of haloperidol 5 mg, diphenhydramine 50 mg, and lorazepam 2 mg for acute agitation as a result. He agreed to give a urine sample on the third day of his initial presentation, and the results were negative. After considering a differential diagnosis of bipolar disorder, schizoaffective disorder, and schizophrenia, based on history, collateral information (diagnosis of ADHD, history of Adderall abuse, seeking different psychiatrists to obtain Adderall, and history of previous hospitalizations/ER visits) from his mother, and clinical presentation, he was given a diagnosis of stimulant-induced psychosis. His behavioral symptoms failed to remit after three days of observation in CPEP, prompting a decision to admit the patient to an inpatient unit under involuntary status for further stabilization based on criteria that he would not be able to take care of himself in that state if he was discharged from hospital.

In the inpatient unit, the patient was initially started on oral risperidone 1 mg twice a day (BID); however, Mr. X continued to refuse medications. Mr. X’s mother was actively involved in the treatment plan. After discussing all the available treatment options in a family meeting in the presence of Mr. X’s mother, Mr. X agreed to take oral Haldol 5 mg BID that was later up titrated to 7.5 mg BID. Mr. X provided reasoning that he had tried risperidone in the past and he did not like how it made him feel. Initially, Mr. X was only partially compliant with oral Haldol, but with repeated counseling and the active involvement of Mr. X’s mother in the treatment plan, he eventually became compliant. Throughout his inpatient stay, he continued to remain insistent on getting Adderall or Vyvanse for his ADHD without specifying dosage. In light of his two inpatient psychiatric hospitalizations in the past six months and his continuation of Adderall abuse as well as non-compliance with prescribed antipsychotic treatment, a clinical decision was made to offer long-acting Haldol to the patient. After discussing with Mr. X and his mother, with the agreement of Mr. X, he was given two injections of Haldol Decanoate 50 mg and 100 mg intramuscularly as per the manufacturer’s guidelines. Additionally, Mr. X was prescribed clonidine 0.1 mg daily for the management of his ADHD. Mr. X tolerated the prescribed treatment without any adverse effects. Toward the end of his hospitalization, he no longer exhibited grandiose delusions of being a supreme court justice. His bizarre and disorganized behaviors were reduced significantly, and he was safely discharged (two weeks of hospitalization) to be followed up in a partial hospitalization program. Following the discharge, he remained compliant with the treatment plan for a follow-up period of six months.

## Discussion

The endogenous sensitization hypothesis suggests that first-episode psychosis patients have a heightened response to acute amphetamine administration, causing excessive dopamine release when compared to healthy volunteers [8]. At the same time, healthy volunteers given amphetamine for a prolonged period of time develop similar sensitization as seen in first-episode psychosis patients [8].

Stimulant medications have been used for decades in the treatment of ADHD with a good safety record when used in the therapeutic dosage range. Additionally, treatment with stimulant medications has been shown to reduce the risk of subsequent illicit substance use by 60% in ADHD patients compared to untreated ADHD patients [1]. With evidence of differential effects of stimulants in ADHD patients, it is possible that sensitization occurring with prolonged stimulant use might be beneficial to these patients, correcting the dopamine deficit in the prefrontal cortical regions of the brain. Whereas in the non-ADHD population, prolonged amphetamine exposure and subsequent excessive dopamine release from amphetamine might make them prone to developing psychotic disorders. The increasing trend of overdiagnosing ADHD and treatment with stimulants may explain the psychotic presentation in healthy individuals after prolonged stimulant use [5,6]. At the same time, epidemiological studies suggest an increasing trend of non-medical Adderall use, primarily obtained from friends and family members, contributing to increased ED visits [9].

Although it remains challenging to separate first onset psychosis from stimulant-induced psychosis that persists, there are some studies that suggest different symptom presentations in the case of amphetamine-induced persistent psychosis. For example, a study by Yang et al. suggests that methamphetamine-induced psychosis is marked by less paranoia and negative symptoms compared to patients with primary schizophrenic psychosis [10].

Besides the obvious predictors of amphetamine-induced psychosis that include the dosage and duration of amphetamine use, some studies have highlighted the additional role of gamma-aminobutyric acid (GABA) dysfunction resulting in a heightened risk of stimulant-induced psychosis. A study done by Ahn et al. suggests that pre-existing GABA deficits increased vulnerability to stimulant-induced psychosis in healthy subjects [11].

Treatment of stimulant-induced psychosis seems to be similar to that of treatment of psychotic disorders and schizophrenia. A recent systematic review by Fluyau et al. found that aripiprazole, haloperidol, quetiapine, olanzapine, and risperidone were able to reduce or control stimulant-induced psychosis with comparable efficacy, and all treatments were well tolerated [12]. A systematic review by Coles et al. found long-acting injectable antipsychotics to be an effective option for the treatment of dual diagnosis schizophrenia and substance use disorder [13].

We described a case of Adderall-induced psychosis that persisted long after a negative urine drug test. We described the diagnostic dilemma as a result. Based on the above-mentioned history, collateral information, and clinical judgment, we diagnosed Mr. X with stimulant-induced psychosis. Although not FDA approved for the treatment of stimulant-induced psychosis, we successfully used a long-acting injectable for haloperidol decanoate in light of Mr. X’s two inpatient hospitalizations within the past six months and history of non-compliance with antipsychotic treatment.

## Conclusions

Adderall, when used above the therapeutic dosage, can lead to psychotic episodes that may persist. There is a pressing need to identify individuals at a higher risk of prescription stimulant abuse. Additionally, further research is needed to identify individuals that are at increased risk of developing persistent psychosis from Adderall abuse. Alpha-2-adrenergic agonists may be a safer alternative for the management of ADHD in such cases. Long-acting Haldol provided a great response in our case; however, further research is required to assess long-acting injectables as a definitive treatment for stimulant-induced psychotic disorders, especially in patients with a history of non-compliance and recurrent hospitalizations.
